# Psychosocial Issues and Experiences of Advanced Stage Lung Cancer Patients Receiving Palliative Care: A Qualitative Study

**DOI:** 10.1002/cam4.71101

**Published:** 2025-07-28

**Authors:** Dilek Yıldırım, Vildan Kocatepe

**Affiliations:** ^1^ Department of Nursing, Faculty of Health Sciences Istanbul Aydin University Istanbul Turkey; ^2^ Department of Nursing, Institute of Health Sciences Izmir Demokrasi University Izmir Turkey

**Keywords:** advanced cancer, lung cancer, palliative care, psychosocial issues, qualitative study

## Abstract

**Background:**

A thorough examination of psychosocial problems and individual patient experiences could contribute to a better understanding of the needs of advanced stage lung cancer patients and the development of personalized care models.

**Methods:**

This qualitative study investigates the psychosocial issues and end‐of‐life coping strategies experienced by advanced‐stage lung cancer patients receiving palliative care at a family health center in Istanbul, Turkey, between January and October 2024.

**Results:**

The average age of the patients was 58.50 ± 14.37 years, and the average duration of diagnosis was 2.156 ± 1.439 years. Most of the patients reported feeling anxiety and fear when they received the diagnosis. They expressed concerns about the impact of the illness on their children, family, and future. Despite the initial anxiety and fears, most palliative care patients with advanced‐stage lung cancer eventually accepted their illness and adapted over time. However, the duration of this adaptation and acceptance varied, and it was observed that in many cases, this acceptance was expressed as something that had to be done out of necessity. All patients had at least three symptoms, with the five most common symptoms being, in order: weakness/fatigue, severe cough, shortness of breath, pain, and weight loss.

**Conclusions:**

While patients emphasized the importance of family support in the coping process, it was observed that issues such as fear of stigma, economic difficulties, and loneliness complicate this process. Feelings of regret and guilt related to a history of smoking were found to be widespread, and these emotions were found to influence the patients' coping and confrontation processes.

## 1 Background

Lung cancer is one of the most common types of cancer worldwide and a leading cause of cancer‐related deaths. The diagnosis is often made at an advanced stage, which limits treatment options and negatively impacts both life expectancy and quality of life [1]. Lung cancer patients are faced not only with physical symptoms but also with significant psychosocial challenges. The diagnosis and treatment of lung cancer can disrupt many aspects of daily life, leading to a loss of self‐awareness, changes in attitudes towards life, personal beliefs, and value systems. This disruption can result in a sense of meaninglessness regarding the continuity and stability of life. Additionally, the side effects of cancer treatment, compounded by the physical and psychological pain experienced by patients, can lead to feelings of helplessness, hopelessness, and a sense of purposelessness [2]. The literature indicates that this patient group experiences higher levels of psychological stress and social challenges compared to patients with other types of cancer. Depression is common and prevalent among lung cancer patients [3], with its prevalence ranging from 19% to 73.9% [4, 5]. For example, the prevalence of depressive feelings is 29.5% in patients with gynecological cancers, while it can reach as high as 73.9% in lung cancer patients. Additionally, the incidence of major depression in lung cancer patients has been found to be 13.1%, which is higher than in many other types of cancer [6].

In recent years, there has been a growing interest in the psychological and social consequences of lung cancer. It is emphasized that psychosocial factors play a critical role in the management of lung cancer, and assessing these factors early on could improve patients' quality of life [7].

A study by Morrison and colleagues (2017) found that approximately one‐third of lung cancer patients experience moderate to severe emotional issues, which are associated with a significant decline in quality of life and an increase in symptom burden. Specifically, patients with advanced‐stage lung cancer reported higher rates of emotional problems. Risk factors include younger age, female gender, smoking, advanced disease, poor performance status, and reduced work capacity. In this context, screening for psychosocial issues at the time of diagnosis and providing individualized psychosocial support approaches are considered crucial components of patient care [8].

Studies conducted with patients in palliative care have revealed that depression and anxiety rates are notably high among lung cancer patients. The prevalence of depression is found to be 43% in patients with small cell lung cancer and 21% in those with non‐small cell lung cancer. Similarly, the prevalence of anxiety is 43% and 25%, respectively. These studies indicate that the level of functional impairment is a significant risk factor for psychosocial issues, and each unit increase on the disability scale is associated with a 41% increase in depression rates [9]. Additionally, the literature highlights that cancer‐related stigma may play a significant role in the development of emotional symptoms in these patients [10].

In international literature, it is often observed that psychological, social, and emotional well‐being in advanced‐stage lung cancer patients is considered a secondary priority in their care processes. For instance, it is noted that psychological issues such as depression, anxiety, and fear of recurrence, as well as social problems like family support, employment, and isolation, are not adequately addressed [11, 12, 13]. However, deficiencies in these areas can negatively impact not only patients' quality of life but also the overall disease management and treatment adherence. This study aims to understand the psychosocial issues and experiences of advanced‐stage lung cancer patients receiving palliative care. A thorough examination of psychosocial problems and individual patient experiences could contribute to a better understanding of the needs of advanced‐stage lung cancer patients and the development of personalized care models.

## 2 Methods

This qualitative study investigates the psychosocial issues and end‐of‐life coping strategies experienced by advanced‐stage lung cancer patients receiving palliative care at a family health center in Istanbul, Turkey. The reporting of this study has been conducted in accordance with the COREQ (Consolidated Criteria for Reporting Qualitative Research) guidelines, which are used for reporting qualitative studies.

### 2.1 Sampling and Setting

The study was conducted between January and October 2024 at a family health center providing home‐based palliative care services in Istanbul, Turkey. In Turkey, healthcare is delivered through a mixed model that encompasses both public and private providers. The vast majority of citizens are covered by the General Health Insurance Scheme, administered by the Social Security Institution (SGK), which ensures access to a broad spectrum of healthcare services, particularly through state hospitals and select private facilities under contract. In recent years, palliative care has become increasingly embedded in the healthcare infrastructure, offered predominantly through oncology departments in tertiary care institutions, community‐based home care programs, and a limited number of dedicated palliative care units. Nonetheless, access to these services is not uniform across the country, with more comprehensive care typically available in urban regions compared to rural areas.

Beyond hospital‐based interventions, home‐based palliative care is supported by the Ministry of Health through structured Home Health Services. These teams provide essential medical care, pain and symptom management, and psychosocial support within the patient's home environment. Additionally, family physicians at Family Health Centers (Aile Sağlığı Merkezleri) are authorized to prescribe medications and issue relevant medical reports for patients receiving care at home. This integrated approach facilitates continuity of care, reduces the need for repeated hospital visits, and supports patients—especially those with advanced illness stages—in remaining in their familiar environments with adequate medical oversight.

The research was carried out with advanced‐stage (Stage III and Stage IV) patients who agreed to participate. Inclusion criteria for the study were individuals aged 18 or older, who were willing to participate, had been diagnosed with lung cancer, were knowledgeable about their disease status, could communicate verbally, and had adequate linguistic expression skills. Patients with a history of other malignancies, those with mental or cognitive disorders, those who were unable to communicate, and those with a language barrier that would prevent them from participating in the interview were excluded from the study.

Potential participants were initially recommended by a nurse and a physician, and then the research team provided detailed information about the study's purpose and content to the participants. Individuals who were willing to participate and met the sampling criteria were included in the study sample. Purposeful sampling was used in this study. In qualitative research, the data collection process is concluded when no new information is being gained, which is considered data saturation [14]. A total of 32 individuals expressed a willingness to participate in the study and were included after signing the informed consent form. The sociodemographic characteristics of the participants and information about their illness are presented in Table 2.

### 2.2 Data Collection

The data were collected through face‐to‐face interviews using a semi‐structured form (Table 1). The semi‐structured form was developed based on a literature review prior to the interviews [2, 15, 16]. The sociodemographic and clinical characteristics of the patients in Table 2 were asked in a question and answer format, not as open‐ended questions. After selecting the participant, the interviewer made the necessary arrangements to ensure that the interview environment was quiet and free from distractions. A suitable day and time for the interview were mutually agreed upon with the participant. During the interview, the interviewer used a voice recorder to capture the content of the conversation. The duration of the interviews varied between 30 and 40 min, depending on the participant's condition. If participants felt fatigued during the interview, it was concluded, and if necessary, another appointment was scheduled to continue the interview or a break was provided.

**TABLE 1:** Interview guide questions.

Number	Interview guide questions
1	How was the period after the diagnosis? What did you experience?
2	How did what you experienced affect you?
3	Were there any challenges or opportunities you wanted to express?
4	How did you cope with these challenges?
5	What were your sources of support during this process?
6	What role did your own character play in this process?
7	Did you experience any changes during this process? If so, how would you compare the changes to before?

**TABLE 2:** Sociodemographic and clinic characteristics of patients (*n* = 32).

Characteristics	(Mean ± SD; range)
Age (years)	58.500 ± 14.375; 20–87
The diagnostic process duration (years)	2.156 ± 1.439; 1–7

	*n* (%)
Gender	
Female	16 (50)
Male	16 (50)
Marital status	
Married	18 (56.3)
Single	14 (43.8)
Educational status	
Illiterate	1 (3.1)
Literacy	8 (25)
Primary school	11 (34.4)
Secondary school	1 (3.1)
High school	8 (25)
University and above	3 (9.4)
Employment status	
Housewife	6 (18.8)
Retired	17 (53.1)
Unemployed	6 (18.8)
Other	3 (9.4)
Self‐perceived income level	
Medium	22 (68.8)
Low	10 (31.2)
Smoking status	
Yes	7 (21.9)
No	3 (9.4)
Quit	22 (68.8)
Alcohol consumption status	
Yes	3 (9.4)
No	18 (56.3)
Quit	11 (34.4)
Disease stage	
Stage III	21 (65.6)
Stage IV	11 (34.4)
Cancer type	
Non‐small cell lung cancer	29 (90.6)
Small cell lung cancer	3 (9.4)
Surgical treatment	
Yes	6 (18.8)
No	26 (81.2)
Chemotherapy	
Yes	32 (100)
Radiotherapy	
Yes	32 (100)
Living situation	
Living alone (in own home)	5 (15.6)
Living with family	27 (84.4)

### 2.3 Data Analysis

The interviews were audio recorded and transcribed verbatim after the participants' personal information was removed. The resulting transcripts were independently reviewed by two academics. The interpretive descriptive approach was used as the theoretical framework to guide the interpretation of the data [17]. Beyond simple descriptive methods, interpretive description adds meaning to the analysis findings and provides insights into how research results can be applied in practice, particularly in fields like nursing, which is a health and practice‐based discipline [18]. The data analysis process was carried out using an inductive approach. This means that the researcher analyzed the data in an open‐minded manner, without biases or assumptions, following a method aimed at discovering internal relationships and characteristics, and making generalizations from individual cases. After the transcripts were read and analyzed, initial categories (open coding) were created, which were later divided into relevant subthemes. The subthemes were organized, combined, and arranged to form larger themes. The results were discussed until consensus was reached among the team members, and any disagreements were resolved.

### 2.4 Ethics Considerations

We obtained approval from the ethics committee of the Committee of Istanbul Aydin University, dated May 04, 2023, and numbered 2023‐04. All procedures performed in this study were in accordance with the ethical standards of the institutional research committee and with the Helsinki Declaration and its later amendments or comparable ethical standards. All participants received a research information leaflet and signed an informed consent form.

## 3 Results

A total of 35 eligible patients were invited, 32 of whom agreed to participate (a 91.4% approval rate), and ultimately, 32 patients completed the interview. The sociodemographic and clinical characteristics of the patients are presented in Table 2. The average age of the patients was 58.50 ± 14.37 years, and the average duration of diagnosis was 2.156 ± 1.439 years. Half of the patients were women (*n* = 16, 50%), and half were men (*n* = 16, 50%). Of the patients, 56.3% were married, 34.4% had completed elementary school, 53.1% were retired, and 68.8% reported a moderate economic level. 68.8% of the patients had previously smoked but had quit, and 56.3% reported not using alcohol. 65.6% of the patients were in stage III, 90.4% of the patients were diagnosed with non‐small cell lung cancer, 81.2% had not undergone surgical treatment, and all of the patients (100%, *n* = 32) had received chemotherapy and radiotherapy. A total of 84.4% of the patients were living with their family members. The remaining 15.6% lived alone in their own homes, though they received regular visits from family members (Table 2).

### 3.1 Main Theme 1: Emotional Reactions to the Diagnosis

#### 3.1.1 Subtheme 1.1: Initial Shock, Fear, and Anxiety

Most of the patients reported feeling anxiety and fear when they received the diagnosis. They expressed concerns about the impact of the illness on their children, family, and future. As stated by the patients:

I was very upset, I wasn't expecting it. I went because I was coughing, they thought it was bronchitis, it started with the cough, then I was told I had cancer. (66 years old male patient)

I became indifferent to life, I felt really bad, everything feels empty and meaningless. I thought about what might happen to me. (57 years old, male patient)

It was anxious, I wasn't really surprised when I learned about my illness. I was just sad for my children. (72 years old, female patient)

### 3.2 Main Theme 2: Coping and Adaptation Process

#### 3.2.1 Subtheme 2.1: Gradual Acceptance

Despite the initial anxiety and fears, most palliative care patients with advanced‐stage lung cancer eventually accepted their illness and adapted over time. However, the duration of this adaptation and acceptance varied, and it was observed that in many cases, this acceptance was expressed as something that had to be done out of necessity. Patients reported that with the support of their families, they accepted the illness and began the treatment process. According to the patients' statements, this process took up to 2 months.

I felt really bad, I thought life was meaningless, I was in shock, it took me time to accept it. (61 years old, male patient)

The initial period was spent in shock and acceptance. This lasted for about 30‐45 days. After accepting it (of course, out of necessity), we continued with the process as normal, with the support of my family. (49 years old, female patient)

### 3.3 Main Theme 3: Physical Challenges and Symptom Burden

#### 3.3.1 Subtheme 3.1: Common Physical Symptoms

Most patients reported that they experienced the most difficulties due to physical problems. All patients had at least three symptoms, with the five most common symptoms being, in order: weakness/fatigue, severe cough, shortness of breath, pain, and weight loss. Patient statements:

I was experiencing weakness, loss of appetite, and shoulder pain. As I lost weight, I felt weak and exhausted. (35 years old male patient)

I experience shortness of breath, coughing, swelling on my face, weight loss, and difficulty walking. (58 years old female patient)

I experience a lot of shortness of breath and coughing. I miss my old health a lot. (62 years old female patient)

### 3.4 Main Theme 4: Psychological Struggles

#### 3.4.1 Subtheme 4.1: Helplessness and Fear of Death

Most patients reported not being psychologically well and feeling a sense of helplessness.

Of course, during this process, I wondered how I could be, I locked myself in my room, I didn't want to talk, I wanted to be alone. Later, I thought, this can't go on like this. (76 years old, male patient)

I am indifferent to life and I am scared. I thought I would die unaware of what was going to happen to me, thinking about death frightened me. (39 years old female patient)

I felt very helpless most of the time… (sighs, takes a deep breath). (72 years old, female patient)

Sadness and helplessness feel like they've stuck to me…. (64 years old, female patient)

#### 3.4.2 Subtheme 4.2: Withdrawal and Desire for Isolation

Some patients preferred being alone and avoided social interactions.

I wanted to distance myself from others and people, to sleep all the time and be alone. I didn't want to hear words like “you will get better” from those around me. (35 years old, male patient)

### 3.5 Main Theme 5: Socioeconomic Difficulties and Stigma

#### 3.5.1 Subtheme 5.1: Financial Burden

Most palliative care patients with advanced‐stage lung cancer reported experiencing economic difficulties. Although their healthcare needs were financially covered under health insurance, the patients expressed that they were unable to work and could not meet their daily financial needs.

We've had a lot of financial difficulties, we're struggling. (crying). (67 years old, female patient)

I went to the health center, we want to get an intravenous treatment. We are facing financial and emotional difficulties outside, at least let them administer an intravenous treatment. (66 years old, male patient)

Patients with cancer, like me, should receive more psychological, financial, and emotional support from the hospital. (65 years old, female patient)

#### 3.5.2 Subtheme 5.2: Social Withdrawal and Internalized Stigma

Some patients, especially younger ones, felt discomfort with others' reactions and withdrew socially.

It bothers me when people wrinkle their faces and look at me with pity when they hear that I have cancer. (35 years old, male patient)

### 3.6 Main Theme 6: Sources of Support and Regret

#### 3.6.1 Subtheme 6.1: Emotional and Social Support

Most of the patients interviewed reported receiving social and emotional support from their families, children, and close friends. A large portion of the patients identified their “doctor” as the source of informational support.

#### 3.6.2 Subtheme 6.2: Regret Over Health Behaviors

Some patients, according to their own descriptions, expressed feelings of regret. They particularly regretted their use of alcohol and cigarettes, believing that they had become ill because they had not taken proper care of their health.

I question my life, I smoked for many years, and I keep thinking that it might be the cause. I feel regret. (58 years old, female patient)

I regret smoking. (51 years old, female patient)

Of course, it happened, and I felt like I was going to die, I felt drained as if life was coming to an end. Before the illness, my alcohol and cigarette consumption was very high, and I thought these had an effect, which made me angry at myself. (37 years old, male patient)

I wish I had paid more attention to my health in the past years, I regret it. (72 years old, male patient)

## 4 Discussion

According to the data collected from lung cancer patients in the study, advanced‐stage lung cancer patients receiving palliative care experience significant physical, psychological, and social challenges from the moment of diagnosis. These patients have emphasized the critical importance of family support during the process of confronting the illness and adapting to it. However, it is apparent that they face issues such as economic difficulties, stigma, and loneliness, and that they require more psychosocial support during this process. Additionally, regret and questioning of past lifestyles play a significant role in the process of reconciliation with themselves. These findings suggest that care models for lung cancer patients should be restructured to address individual needs.

Receiving a serious diagnosis like lung cancer not only has physical effects but also creates significant psychosocial impacts on the individual. In the present study, most patients reported experiencing anxiety and fear upon diagnosis. Additionally, patients expressed concerns about how the disease would affect their children, families, and future. A lung cancer diagnosis is often associated with uncertainty, fear of death, and a significant deterioration in quality of life. These emotions are central to the palliative care process from the moment of diagnosis. The diagnosis of a life‐limiting illness often leads to significant changes in how a person experiences and understands life and death [19].

The literature includes studies indicating that anxiety levels are high in such patients, and these studies emphasize the need for psychosocial support [8, 9, 20].

In a study by Zhang et al. (2022), it was stated that lung cancer can be perceived as a highly threatening illness that alters a person's self‐perception and leads patients to deep reflections on the meaning of life [2]. After receiving the diagnosis, patients experience a sense of uncertainty about the future [21], struggle with the fear of death, and tend to avoid discussing their illness. Furthermore, by becoming aware of their past unhealthy lifestyles and the burden the disease places on their families, they may experience intense feelings of shame and guilt. The potential impact of the illness on patients' children and families extends beyond the individual level, deeply affecting family dynamics as well. This situation can trigger feelings of guilt, anxiety, and fears about the future. Patients may fear not only the effect of the illness on their physical condition but also the potential consequences for the future of their loved ones. This can create a significant stress factor, combining the loss of social roles and independence.

In the present study, it was observed that advanced‐stage lung cancer patients receiving palliative care experienced intense anxiety and fear at the time of diagnosis, but over time, they tried to cope with these emotions and achieved acceptance and adaptation to the illness. However, this process occurred over varying periods for each patient, with some patients taking up to 2 months. The literature indicates that advanced‐stage cancer patients tend to gradually accept the emotional burden they experience at the onset of the illness and develop awareness of death over time [22, 23]. This process of acceptance facilitates the patients' adaptation to the illness both psychologically and socially.

Research shows that patients develop various strategies to cope with uncertainty. Strategies such as avoidance, maintaining normalcy, minimizing the impact of the illness, and focusing on the current treatment have been identified as important mechanisms in coping with the stress caused by the illness [24]. However, it is emphasized that for these strategies to be used effectively, patients need to understand the disease process and actively cope with it [25]. However, in the present study, the preference of most patients to be alone suggests that this may have limited their ability to develop coping strategies. The statements of patients who preferred to be alone indicate that this preference could be interpreted more in terms of avoidance and social isolation. This suggests that the patients chose to withdraw rather than share their emotional burdens, and this preference may have reduced their level of social support. However, the literature emphasizes that social support and emotional sharing are crucial for enhancing the psychological well‐being of cancer patients [25]. It should be determined whether the preference for solitude is a coping strategy or the result of helplessness and isolation. Healthcare professionals, by understanding the tendency of these patients towards loneliness, can develop appropriate intervention methods and contribute to the development of more effective coping mechanisms during the disease process.

In the present study, it was observed that a significant portion of advanced‐stage lung cancer patients experienced feelings of regret and guilt due to their past smoking habits. Similarly, the study by Zhang et al. (2022) found that a significant number of lung cancer patients had a history of smoking, and this was found to trigger feelings of self‐blame and guilt in these patients [2]. It was particularly understood that patients believed unhealthy lifestyle choices, such as smoking, contributed to their illness, and that this situation had negative effects both individually and socially.

Fear of stigma and the psychosocial issues arising from it are commonly observed in lung cancer patients. The literature reveals that a large majority of lung cancer patients (95%) experience stigma, which stems from negative attitudes towards smoking and perceptions related to the illness itself [26]. Stigma not only leads patients to self‐blame but also results in their isolation from their environment and social life. There is cross‐sectional and longitudinal evidence suggesting that this isolation exacerbates health‐related negative outcomes such as anxiety, depressive symptoms, and reduced quality of life [27]. Stigma related to smoking causes serious psychosocial issues in lung cancer patients, and these issues affect not only the individual's health condition but also the patients' treatment process and social relationships.

The findings of this study suggest that implementing structured psychosocial support interventions, such as counseling services, peer support mechanisms, and personalized coping strategies, may significantly improve the emotional well‐being of patients receiving palliative care. Integrating routine screening for psychological distress—including anxiety, depression, and fear of death—into palliative care protocols could also facilitate early identification of at‐risk individuals and timely referral to appropriate mental health services. In addition, strengthening community‐based palliative care and home care programs can help maintain continuity of psychosocial support beyond the hospital environment, particularly for patients with limited mobility or those preferring to remain at home.

### 4.1 Limitations of the Study

The study was conducted with a limited number of participants from a single healthcare center. The findings may not be generalizable to different demographic groups or cultural contexts. During the data collection process based on interviews, patients may have struggled to express certain emotions, which could have limited the depth of the data. The study did not track the long‐term changes in patients' illness trajectories. Longer‐term and follow‐up studies are needed. Despite these limitations, the present study sheds light on the challenges faced by advanced‐stage lung cancer patients and provides valuable insights that can guide future research in this field. This study did not include objective clinical data such as antidepressant use or the length of stay in palliative care, which may have further enriched the interpretation of patients' psychosocial experiences. Future studies may benefit from combining qualitative findings with selected clinical parameters to provide a more comprehensive perspective.

## 5 Conclusions

In the present study, it was determined that advanced‐stage lung cancer patients receiving palliative care experience intense physical, psychological, and social issues from the moment of diagnosis. While patients emphasized the importance of family support in the coping process, it was observed that issues such as fear of stigma, economic difficulties, and loneliness complicate this process. Feelings of regret and guilt related to a history of smoking were found to be widespread, and these emotions were found to influence the patients' coping and confrontation processes. Furthermore, most patients preferred to be alone, and it was assessed that this might result in insufficient utilization of social support. Palliative care should adopt a patient‐centered, empathetic, and multidisciplinary approach to address such fears. Additionally, integrating individual and family‐focused interventions with psychosocial support programs may improve care outcomes. This approach can reduce patients' anxiety, enhance their quality of life, and strengthen their coping mechanisms. Education and information programs should be implemented to support the active participation of families in the disease process. Family members should be guided on how to support the patient during the course of the illness.

In conclusion, enhancing the quality of life in patients with advanced‐stage lung cancer requires a multidimensional approach that goes beyond physical symptom management. Incorporating mental health screening into standard palliative care, improving access to psychological counseling, and expanding the reach of community‐based support services are essential steps in addressing the complex psychosocial needs of this patient population.

## Author Contributions

Dilek Yildirim was involved in conceptualization, data curation, formal analysis, funding acquisition, project administration, methodology, software, visualization, writing – original draft, and writing – review and editing. Vildan Kocatepe was involved in conceptualization, data curation, formal analysis, funding acquisition, methodology, visualization, writing – original draft, and writing – review and editing.

## Ethics Statement

We obtained approval from the ethics committee of the Committee of Istanbul Aydin University, dated May 04, 2023 and numbered 2023‐04. All procedures performed in this study were in accordance with the ethical standards of the institutional research committee and with the Helsinki Declaration and its later amendments or comparable ethical standards. All participants received a research information leaflet and signed an informed consent form.

## Consent

Informed consent was obtained from all individual participants included in the study.

## Conflicts of Interest

The authors declare no conflicts of interest.

## Data Availability

Data are available upon request from the corresponding author.
